# Targeted KRAS Mutation Assessment on Patient Tumor Histologic Material in Real Time Diagnostics

**DOI:** 10.1371/journal.pone.0007746

**Published:** 2009-11-04

**Authors:** Vassiliki Kotoula, Elpida Charalambous, Bart Biesmans, Andigoni Malousi, Eleni Vrettou, George Fountzilas, George Karkavelas

**Affiliations:** 1 Department of Pathology, Papageorgiou Hospital, Aristotle University of Thessaloniki, School of Medicine, Thessaloniki, Greece; 2 Department of Medical Informatics, Papageorgiou Hospital, Aristotle University of Thessaloniki, School of Medicine, Thessaloniki, Greece; 3 Department of Medical Oncology, Papageorgiou Hospital, Aristotle University of Thessaloniki, School of Medicine, Thessaloniki, Greece; 4 Department of Human Genetics, University of Leuven, Leuven, Belgium; Bauer Research Foundation, United States of America

## Abstract

**Background:**

Testing for tumor specific mutations on routine formalin-fixed paraffin-embedded (FFPE) tissues may predict response to treatment in Medical Oncology and has already entered diagnostics, with KRAS mutation assessment as a paradigm. The highly sensitive real time PCR (Q-PCR) methods developed for this purpose are usually standardized under optimal template conditions. In routine diagnostics, however, suboptimal templates pose the challenge. Herein, we addressed the applicability of sequencing and two Q-PCR methods on prospectively assessed diagnostic cases for KRAS mutations.

**Methodology/Principal Findings:**

Tumor FFPE-DNA from 135 diagnostic and 75 low-quality control samples was obtained upon macrodissection, tested for fragmentation and assessed for KRAS mutations with dideoxy-sequencing and with two Q-PCR methods (Taqman-minor-groove-binder [TMGB] probes and DxS-KRAS-IVD). Samples with relatively well preserved DNA could be accurately analyzed with sequencing, while Q-PCR methods yielded informative results even in cases with very fragmented DNA (p<0.0001) with 100% sensitivity and specificity vs each other. However, Q-PCR efficiency (Ct values) also depended on DNA-fragmentation (p<0.0001). Q-PCR methods were sensitive to detect ≤1% mutant cells, provided that samples yielded cycle thresholds (Ct) <29, but this condition was met in only 38.5% of diagnostic samples. In comparison, FFPE samples (>99%) could accurately be analyzed at a sensitivity level of 10% (external validation of TMGB results). DNA quality and tumor cell content were the main reasons for discrepant sequencing/Q-PCR results (1.5%).

**Conclusions/Significance:**

Diagnostic targeted mutation assessment on FFPE-DNA is very efficient with Q-PCR methods in comparison to dideoxy-sequencing. However, DNA fragmentation/amplification capacity and tumor DNA content must be considered for the interpretation of Q-PCR results in order to provide accurate information for clinical decision making.

## Introduction

Based on accumulated knowledge about tumor biology, newer drugs are meant to treat cancer in a more rational way than classic chemotherapy, i.e., by targeting specific molecules and pathways that are essential for promoting tumor growth, maintenance and metastasis. In this context, EGFR, a HER family receptor tyrosine kinase, has emerged as a major molecular target. Because EGFR was considered to be involved in the pathogenesis of most epithelial cancers [1], anti-EGFR drugs were anticipated to improve outcome for millions of patients worldwide. In fact, though, these drugs dramatically benefit only a small percentage of cancer patients, based on the alterations concerning EGFR itself (e.g., specific mutations targeted by small molecule tyrosine kinase inhibitors [TKIs]) or molecules in the EGFR effector pathways (for example, KRAS [official gene name: v-Ki-ras2 Kirsten rat sarcoma viral oncogene homolog; aliases: KRAS2, RASK2] mutations hampering therapeutic EGFR antibodies and possibly TKIs as well). Of note, success rates of as low as 5% correspond to hundreds thousands of patients worldwide for the major cancer types (breast, lung, colorectal). Hence, if aiming in a rational and beneficial use of molecule targeting drugs it is necessary to identify patients who will truly benefit from such treatments, thus limiting unnecessary toxicities, treatment delays [2], [3] and health care costs [4].

Patient selection is required for drugs that are labeled for a certain molecular target when approved for clinical practice. A recent development in this context concerns two anti-EGFR antibodies, cetuximab and panitumumab, that have been labeled for use in metastatic colorectal cancer (CRC) under the condition that the tumor carries a wild-type KRAS gene [5], [6]. This decision was based on accumulating evidence showing that CRC patients with KRAS mutant tumors do not benefit from treatments with anti-EGFR antibodies [7], [8], [9], [10], [11]. Hence, patients with mutant KRAS tumors are not eligible for treatment with these drugs; the clinician must have the information on KRAS mutation status of the tumor when assessing the patient; the information provided for clinical decision making must be accurate.

The recent 10-year history of diagnostic predictive tests on solid tumors shows that successful patient selection for molecularly targeted drugs is based on three main parameters: (a) biological relevance of the molecular marker, (b) methodology chosen for the investigation of the marker, and (c) template characteristics.

Starting with the latter, diagnostic tests for the assessment of any marker at any molecular level (e.g., proteins – immunohistochemistry, mutations – DNA) are performed on routine diagnostic tumor material, i.e., formalin-fixed paraffin-embedded (FFPE) tumor tissue containing molecular templates suffering from protein cross-linking with formaldehyde. The consequences of fixation, embedding and extraction methods on nucleic acid retrieval from FFPE tissues have well been recognized and described [12], [13], [14], [15], [16], whereby formalin fixation may degrade nucleic acids –a serious problem- but it also deactivates their destructing nucleases –a stabilizing effect. Thus, while molecular templates from FFPE tissues are of inferior quality as compared to their frozen counterparts, these may still be useable for a lot of recently developed methods for nucleic acid investigation, even microarray profiling and wide genome scans, while the main advantage from using molecular FFPE templates is accurate correlation of results with tissue histology.

In regard to the first two parameters, although KRAS mutation status is, in fact, a negative marker predicting for resistance, its biological relevance seems well established: about 30–40% of CRC carry mutations in this gene occurring early in colorectal carcinogenesis [17], [18], [19]. When present, KRAS mutations result in the constitutive activation of this EGFR signaling pathway thus rendering attempts of blocking the extra-cellular part of this receptor in vain [1]. The majority (>98%) of KRAS mutations in CRC are point mutations located in two neighboring codons, 12 and 13, and can thus easily be targeted with PCR-based methods. A variety of PCR methods, increasingly real time PCR (Q-PCR) targeted mutation detection assays, are currently applied for diagnostic KRAS mutation assessments [20], [21], some of which have already acquired the license for in vitro diagnostic (IVD) use in Europe [22]. These methods are developed to overcome the shortcomings of conventional dideoxy-sequencing, the as yet golden standard for mutation assessment, namely labor, time and expertise requirements, low sensitivity at 30% detection of mutant cells in tissues and variously low efficiency on FFPE-DNA [23], [24]. Indeed, Q-PCR methods are easy, fast and surpass the requirement of “1% sensitivity” set for diagnostic mutation assessments [21], while the infrastructure necessary to perform these tests is increasingly acquired in diagnostic laboratories. Of note, 1% sensitivity mostly concerns 1% selectivity, as explained recently [25], while the 1% cut-off is set arbitrarily. At present, although Q-PCR methods are in general more efficient than dideoxy-sequencing on FFPE material, since they target small sequences likely to be preserved upon formalin fixation, it is still not clear how they perform on a daily routine basis in this context.

In comparison to research, where mutation investigations have long been applied on FFPE-tissues, mutation assessment in the diagnostic setting is distinctive in that (a) it is practically impossible to interfere with general pathology practice issues, such as surgical material processing or fixation and paraffin block storage conditions, as suggested for research purposes [13], because the FFPE material submitted to a molecular lab has usually been originally processed elsewhere, and (b) the end-point is not statistical significance upon comparison of different parameters but a result that will aid in clinical decision making for the individual patient. Because of (a), tumor cell content and sample DNA degradation are parameters that can not be changed for a submitted diagnostic material; because of (b), diagnostic samples with “unfavorable” DNA quality or low tumor cell content can not be lightly excluded from analysis.

Herein, we report on our experience on prospective diagnostic KRAS mutation testing with three methods including dideoxy-sequencing that was used as the reference method for mutation validation, and two Q-PCR methods, the IVD DxS-KRAS test and a Taqman-minor-groove-binder (Taqman-MGB, TMGB) test that was standardized and validated in our lab, by taking into consideration not only the efficiency of the methods but mostly focusing on template characteristics.

## Methods

### Tissue specimens and processing

A total of 210 FFPE CRC specimens were evaluated in this study upon permission from the Medical Ethics Review Board (A9586/21-5-08), School of Medicine, Aristotle University Thessaloniki (AUTH). All patients had signed an informed consent form for the use of their biologic material for diagnostic and research purposes. CRC specimens (Table 1) were derived from the following sources:

**Table 1:** FFPE sample characteristics in the diagnostic (A) and in the unfavorable control group (B).

	group A	group B	
**block age (210 cases)**			
all samples	135	75	
2005–2008	114	29	
before 2005	21	46	
**percentage of tumor cells**			
>30%	105^&^	75^&^	
>10–30%	24	none	
∼10%	6	none	
**DNA quantity (ug/ml)**			
mean	81,7	67,4	
range (min–max)	12,3 to 383,5	12,5 to 147,3	
**A260/280 ratio**			
mean	1,67	1,27	
range (min–max)	1,21 to 1,98	1,18 to 2,6	
1,6–1,8 (n samples)	94/135	2/75	
**DNA fragmentation test (n samples)**			
≥300 bp (less fragmented)	102 (75,6%)	none	
100 & 200 bp (intermediate)	17 (12,6%)	2	
100 bp or no product (heavily fragmented)	16 (11,8%)	73	
**DNA control curve Ct**			
**KRAS-TMGB (∼50 ng/reaction)**			
n samples tested	135	75	
mean*	28,46	34,06	p<0.0001
±SD*	2,71	2,07	
range (min–max)	23,73 to 36,61	30,58 to 39,37	
Ct ≤29** (n samples)	52 (38,5%)	0	
Ct ≤33# (n samples)	128 (94,8%)	26 (34,6%)	
**DxS-KRAS (∼50 ng/reaction)**			
n samples tested	29	66	
meanˆ	31,61	36,60	p<0.0001
±SDˆ	3,49	2,78	
range (min–max)	26,21 to 39,49	31,6016 to 40	
Ct ≤29** (n samples)	8 (27,5%)	0	

&  =  >50% tumor cells for 88 samples in group A and all samples in group B

* =  values from all samples (n = 210 for this test) for the DNA control assays employed (7 assays)

** =  Ct (Cycle threshold) limiting value for reliable highest sensitivity assessments with each test (<0.7% mutant tumor cells with the KRAS-TMGB and <1% with the DxS-KRAS test, respectively)

#  =  Ct value for reliable assessments with a sensitivity of ∼7% tumor cells

ˆ  =  values from all samples tested (n = 95 for this test) for the DNA control assay employed (1 assay)

Group A (n = 135) included material from patients with metastatic CRC that was prospectively investigated for KRAS codon 12 and 13 mutations to predict patient eligibility for treatment with anti-EGFR antibodies, according to current guidelines [5], [6]. Samples in this group included paraffin blocks from previously diagnosed (2001–2008) histologic material that were referred for KRAS mutation testing to the Molecular Lab of the Department of Pathology, AUTH. Tissue processing and original histologic diagnoses had been undertaken in various pathology labs in Greece. Because in our country the processing practice for colectomy specimens does not include buffered formalin, tissue fixation had been accomplished in the majority of cases in simple formalin (∼4% formaldehyde) for various time points. Small specimens, such as punch or needle biopsies, were usually fixed in buffered formalin. Overall, it was impossible to interfere with- and to obtain detailed information on fixation conditions for the majority of the tissues examined.

Paraffin blocks derived from colectomy specimens (n = 98), excision of metastatic sites (n = 29, out of which 13 in the liver, 8 in the lung and 8 in other sites), and punch or needle biopsy specimens from the primary tumor or from metastatic sites (n = 8). In all cases, histological confirmation for the presence of the previously diagnosed tumor was performed by a pathologist, who also marked the most dense tumor area on the H&E section avoiding as much as possible necrotic and hemorrhagic areas and extracellular mucous aggregates. These areas were subsequently macrodissected with a scalpel from thick (10 um) deparaffinized sections and brought into 1.5 ml tubes for tissue digestion. The number of sections used per case (3–8) varied according to the dissected area and to tumor histology, necessitating less for more compact tumors.

Group B (n = 75) included archived FFPE material from patients with CRC from the Dept of Pathology, AUTH. This material derived from colectomy specimens in 74 cases and in one case from biopsy material from a local recurrence. Tumor areas from these specimens had been carefully selected by a pathologist to contain 50–90% neoplastic cells and were placed on TMAs (1,5 mm core diameter). TMA core sections were used for DNA extraction (in total, 30 um thickness per case). From each such case 2000–3000 cells were anticipated. This group was used to assess limiting FFPE-DNA sample requirements for mutation analysis with different methods (unfavorable control group).

### Anticipated tumor DNA content in FFPE tissue extracts

Samples containing >70% tumor cells, as estimated on a 2 um thick H&E section are considered optimal for KRAS genotyping [21]. Since genotyping is performed on genomic DNA, the nuclei of these cells are of interest. One 10 um thick section from such a specimen, however, would correspond to <70% tumor nuclei, since these are >10 um and disoriented (described in models for FISH determinations [26]). By contrast, intact normal cell nuclei are likely to be contained in such a section, since these are substantially smaller in size. Hence, including >2 thick sections will further change and eventually lower the percentage of tumor DNA in the extract, even if an after-dissection H&E retains the original analogies. In the same sense, for a specimen assessed to contain 30% or 10% tumor cells on an H&E section, the anticipated tumor DNA content in the extract will be unpredictably different (lower, most probably). Thus, the histological assessment of tumor cell percentage on an FFPE section can serve only as a very rough estimate for tumor DNA content in the corresponding extract. The approach is still necessary, however, especially in samples with low tumor cell content, in order to evaluate genotyping results with different methods. The percentages shown in Table 1 are H&E estimates.

The above also justify the necessity for tumor cell enrichment for genotyping assessments from FFPE tissues. Macrodissection as described here and elsewhere [7], [27] may be more suitable for KRAS testing than the discussed detailed laser microdissection or needle core dissection [20], because of the long proposed CRC heterogeneity in KRAS mutation status [28], [29] prompting for extensive tumor area sampling.

### DNA extraction and evaluation

**Figure 1:**
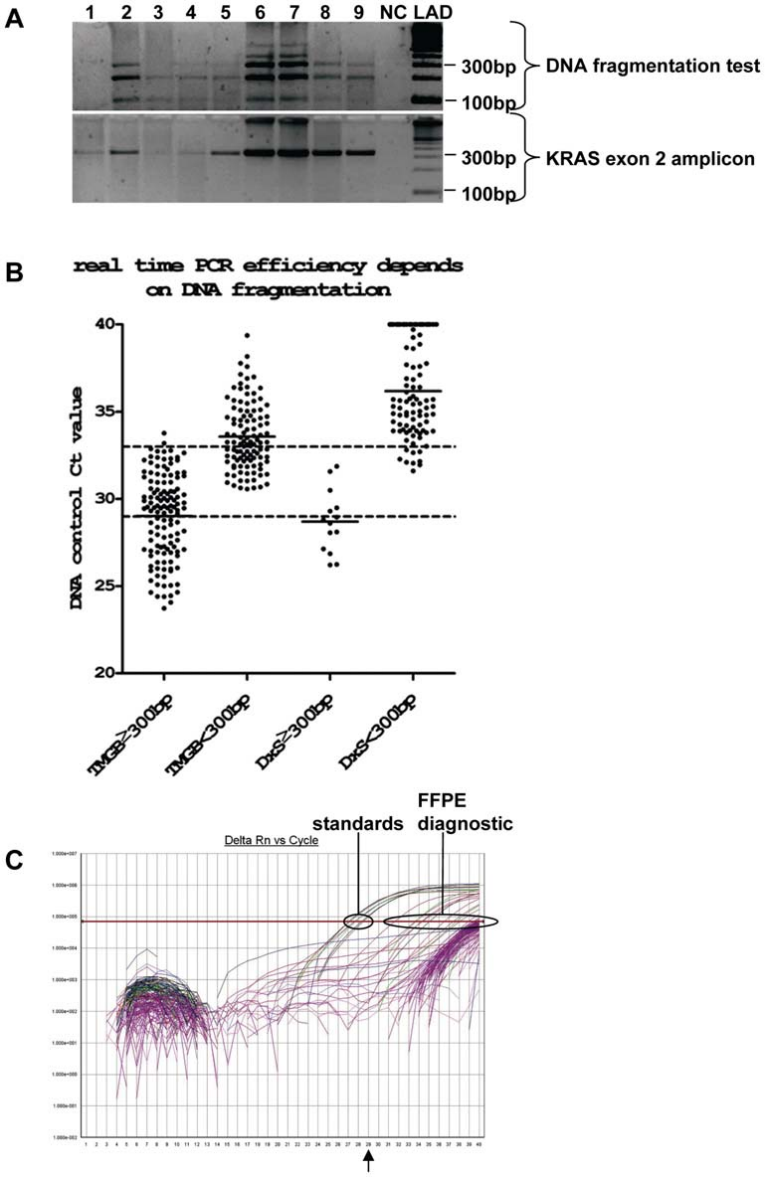
FFPE DNA fragmentation and real time PCR method performance. **A:** A typical series of diagnostic samples tested with the multiplex PCR assay for DNA fragmentation and KRAS exon 2 (intron spanning amplicon). Samples positive for products ≥300 bp are considered as good quality samples yielding the 340 bp product for exon 2 KRAS sequencing. In lanes 1, 3 and 4, faint KRAS product bands could be obtained from samples with very fragmented DNA but the corresponding capillary electropherograms were usually non-informative. In **B**, real time PCR efficiency largely depends on DNA fragmentation. DNA control Ct values reflect the amplification efficiency of FFPE DNA. With both TMGB and DxS-KRAS assays, Ct values from good quality samples were substantially lower than Ct values from very fragmented samples. The majority of FFPE DNA samples yielded DNA control Ct values between 29–33 (dotted lines). In **C**, typical results from diagnostic samples with DxS-KRAS. DNA control Cts for the standards contained in the kit are below 29 (arrow), but corresponding values for the diagnostic samples are >29.

DNA was extracted manually with the QIAamp DNA mini kit [Qiagen, Hilden, Germany], according to the manufacturer's instructions. When necessary (biopsy and metastatic material, mostly), glycogen was added into the lysates as DNA carrier, and the final elution volume was reduced to half in order to obtain more condensed DNA. Concentration (ug/ml) and absorbance (A260/280 ratio) were measured in a UV spectrophotometer [BioPhotometer, Eppendorf, Hamburg, Germany]. Fragmentation of the samples was assessed with the multiplex DNA control assay from BIOMED2 [30], which includes testing for 5 different DNA targets [100, 200, 300, 400 and 600 bp] in the same reaction. Products were visualized on 2% agarose gels (Figure 1A). The same assay was also used to assess FFPE-DNA integrity for array-comparative genomic hybridization (aCGH) [31], while similar testing is required before processing FFPE-DNA samples for wide genome scans, for example, with Affymetrix platforms [http://165.193.231.7/support/technical/technotes/copynumber_ ffpe_technote.pdf].

### Dideoxy-Sequencing for KRAS exon 2 (coding exon 1)

Primers located at intron 1 (forward: 5′-tggtggagtatttgatagtgtattaac-3′) and intron 2 (reverse: 5′-cccaaggtacatttcagataactt-3′) spanning the entire exon 2 were used. PCR products (340 bp) were always visualized on agarose gels and archived prior to sequencing. Sense and antisense sequencing was performed in a 10 ul reaction with the Big Dye Teminator kit v.1.1 [Applied Biosystems, Foster City, USA]. Sequences were visualized upon capillary electrophoresis in an ABI3130 genetic analyzer, initially called with the Sequencing Analysis software and further analysed with the SeqScape software v2.5 [Applied Biosystems/Biosolutions, Athens, GR]. All samples (groups A and B) were submitted to PCR for dideoxy-sequencing.

### KRAS Allelic Discrimination with Taqman-MGB assays (KRAS-TMGB)

Minor grove binder (MGB) modifications at the 3′-end of sequence specific oligos provide the advantage of designing short probes with higher melting temperature (Tm) and increased duplex stability and specificity in comparison to conventional Taqman probes [32], allowing for multiplexing and specific mismatch identification as in the case of single nucleotide polymorphisms (SNPs) [33]. The sensitivity and efficiency of SNP-testing with Taqman-MGB probes in low-medium throughput analyses has been well documented with good quality molecular templates (DNA from peripheral blood, mostly) for germline allelic discrimination or for strain identification of various inflammatory agents [33], [34], [35], [36], [37], whereby these probes do not differ in performance from LNA-probes [38]. A Taqman-MGB assay has been shown as a very reliable alternative to sequencing in identifying the BRAF V600E mutation in CRC FFPE samples [39].

KRAS-TMGB assays were designed with the Primer Express software v1.3 [Applied Biosystems] to detect the 7 more common KRAS mutations in CRC (in alphabetical order, GLY12ALA [G12A], GLY12ARG [G12R], GLY12ASP [G12D], GLY12CYS [G12C], GLY12SER [G12S], GLY12VAL [G12V], GLY13ASP [G13D]). The amplicons were set at 80 bp and the detected sequence was: AGGCCTGCTGAAAATGACTGAATATAAACTTGTGGTAGTTGGAGCTGGTGGCGTAGGCAAGAGTGCCTTGACGATACAGC (codons 12 and 13 are underlined). Samples were run in duplicates of 20 ul reactions, each containing one VIC labelled probe for the wild-type allele (endogenous DNA control serving simultaneously as control for the amplification capacity of the sample) and one specific FAM labelled probe for the mutant allele (target). In the beginning of the application of this assay, sequencing validated mutant samples were included in the runs as controls for the amplification of the mutant allele. Evaluation runs for DNA input and cut-off (sensitivity) assessments were performed in triplicates.

### Rationale for the evaluation of targeted mutation calling with Q-PCR

As instructed by the manufacturer [Applied Biosystems] and as described for the evaluation of Taqman-MGB SNP genotyping in FFPE tissues [40], the presence of genotype variants is called upon plate reading by the software. This may be useful for germline inherited polymorphisms, where heterozygous alleles would be expected to occur in equal amounts. Somatic single-base substitution mutations like the ones found in human tumors in KRAS codons 12 and 13 correspond to SNPs for the cells bearing these mutations, which will appear as heterozygous by any testing (1∶1 ratio for the mutant vs wild-type alleles in mutant cells). In the tissue context, however, the ratio of mutant vs wild-type alleles depends on the presence of non-mutant cells in the sample and would practically correspond to half the percentage of mutant tumor cells. For example, in a sample containing 50% tumor cells, provided that all of them were KRAS-mutant, the mutant alleles to be amplified would correspond to 25% of all alleles included in the sample, which is below the limit of heterozygous calling by the SDS software. Hence, the evaluation of KRAS mutation status was based on the cycle thresholds (Cts) of the wild-type and mutant amplification curves and on the differences between these Cts (dCts), whereby dCtA  =  (Ct for mutant target A) - (Ct for DNA control coamplifying with mutant target A). For each mutant target the respective coamplifying control was used in dCt.

### External validation of KRAS-TMGB results

The same KRAS-TMGB assay was independently developed previously [10] and used for KRAS mutation assessment in CRC [7]. The test is currently used with identical primer/probe sequences for 6 assays and with modified probe sequences for the GLY12ALA assay in the Dept Human Genetics, Lab of Digestive Oncology, University of Leuven. In order to further validate our results with KRAS-TMGB, 106/135 of our diagnostic samples (group A) were assessed for the presence and type of KRAS mutations by the lab in Leuven.

### Therascreen KRAS kit (DxS-KRAS)

This KRAS mutation detection system [DxS, Manchester, UK] uses Scorpion primers designed specifically to recognize the mutated sites (amplification refractory mutation screening [ARMS]) with high efficiency and specificity. The DxS-KRAS kit has been approved as IVD-device by the EMEA (European Medicines Agency) for the identification and typing of the 7 most common KRAS mutations [22]. For the purposes of this study, this system was retrospectively used on 95 samples, following analysis with sequencing and KRAS-TMGB. Samples were run in 25 ul reactions in the ABI7500 real time PCR system described above. The threshold for analysis was set manually in the middle of the DNA control amplification curve, separately for each run, according to the manufacturer's instructions, and dCt values were obtained for (mutant target curve Ct) - (DNA control curve Ct). No template controls and the standards provided by the manufacturer were used in order to monitor results.

### Result presentation and statistics

Statistics (descriptive, chi square, Mann-Whitney and Spearman's correlation tests) were performed by using the SPSS v14 and the GraphPad Prism v5 software; the latter was used for graph presentations as well.

Colormaps of the relative quantification values were built using MATLAB scripts in order to present dCt profiles for all cases studied. Specifically, dCt values were normalized and approximations to −2 for the lower values and 5 for the highest were applied in order to highlight the differences of the quantification values in the mid-region.

## Results

### DNA sample characteristics from FFPE CRC tissue material

DNA sample characteristics are shown in Table 1. In the majority of cases, it was possible to obtain >30% tumor cells in the DNA sample, a threshold set for dideoxy-sequencing sensitivity/selectivity in tissues [21]. However, this threshold could not be reached in 30 diagnostic samples (22%).

As measured with UV-spectrometry, values corresponding to DNA quantity did not differ between groups A and B (Table 1); the samples of the two groups, however, differed substantially in the obtained absorbance values (A260/280 ratios), which correspond to DNA purity (Mann-Whitney p<0.0001). Further, samples in group B appeared heavily degraded in comparison to those in group A (p<0.0001), as observed by their performance with the multiplex PCR fragmentation test (Table 1). If comparing all samples, the efficiency of the fragmentation test was related to the A260/280 ratios and to block age (Mann-Whitney p = 0.003 and p = 0.010, respectively), in line with previous observations for continuing nucleic acid degradation after fixed tissue embedding [41].

In all samples yielding ≥300 bp products with the fragmentation test, amplification of single PCR targets for sequencing was also successful; in addition, in some cases with very fragmented DNA, single 340 bp KRAS products were obtained (Figure 1A). However, sequencing was not always informative in these cases (Table 2).

**Table 2:** Efficiency of the three methods tested for KRAS mutation analysis on FFPE-DNA samples from the diagnostic group A and the unfavorable control group B.

	eligible		informative	
**KRAS-TMGB**	210		209	99,5%
group A	135		135	**100%**
group B	75		74	98,7%
fragmentation (≤200 bp)	108		107	**99,1%**
**Cycle sequencing**	129/210*	61,5%	111/210	52,8%
group A	129/135	**95,5%**	111/135	**82,2%**
group B	0/75		na	na
fragmentation (≤200 bp)	7/108	**6,5%**	2/108	**1,9%**
**Therascreen KRAS**	95		65	68,4%
group A	29		28	**96,6%**
group B	66		37	56,1%
fragmentation (≤200 bp)	81		51	**62,9%**

* =  samples were eligible for sequencing when the corresponding PCR product was visualized upon agarose gel electrophoresis.

na  =  not applicable.

### The efficiency of dideoxy-sequencing and Q-PCR methods on FFPE-DNA samples depends on DNA fragmentation

Sequencing efficiency corresponds to informative nucleotide (base) calling in the electropherograms obtained by the software employed in each case. Sequencing efficiency was inversely related to the degree of DNA fragmentation in both groups (p<0.0001), and to a lesser extent to the A260/280 ratios in group A (p = 0.008) and to DNA input (p = 0.032). The efficiency of this method is shown in Table 2. Yielding the 340 bp KRAS product, as observed upon agarose gel electrophoresis (Figure 1A), conferred to the samples eligibility for further processing for sequencing (86,1% informative results from eligible samples). In 28/135 cases (20.7%) we repeated the whole procedure (DNA extraction and PCR) in order to obtain better quality templates. This attempt yielded PCR products and interpretable electropherograms in only 4 additional cases.

The efficiency of Q-PCR methods is reflected in the Ct (cycle threshold) values obtained for the DNA control target included in each test, whereby (a) the higher the Ct the lesser amplifiable DNA in the sample, and (b) appropriate setting of the reading threshold is very important when using open systems, such as the ABI7500 instrument [http://www3.appliedbiosystems.com/cms/groups/mcb_marketing/documents/generaldocuments/cms_042502.pdf]. Usually, Q-PCR assays are reported to yield Cts <29 for DNA amounts of 10 ng or less. These guidelines, however, do not seem to apply to FFPE-DNA templates. In our hands, the amount of 10 ng DNA input per reaction as suggested by the manufacturer (DxS) in order to obtain DNA control Cts ∼29 was not sufficient to obtain this Ct value. By increasing DNA input to 50 ng/reaction, the desired Ct 29 value was obtained in some cases (Table 1), greatly depending on DNA fragmentation (Figure 1B). The same was observed with KRAS-TMGB as well, (Table 1, Figure 1B). The majority of diagnostic samples and all samples with unfavorable DNA quality yielded Cts>29, performing much worse than the artificial template standards included to test for method performance in the DxS kit (Figure 1C).

**Figure 2:**
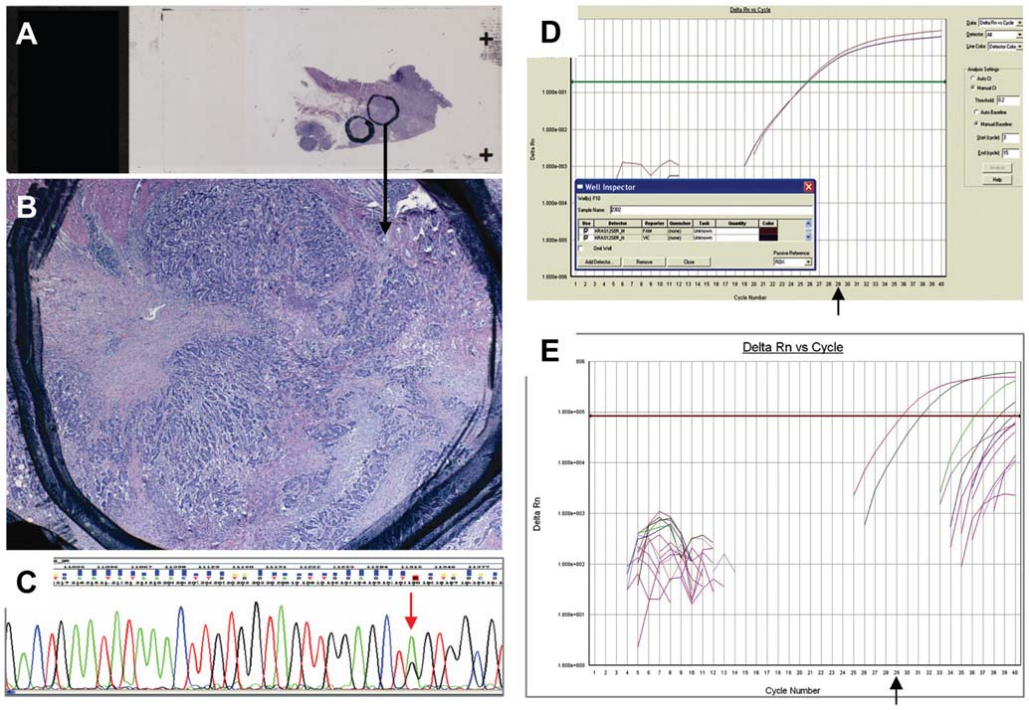
Performance of a typical good quality FFPE-DNA diagnostic sample with the three methods applied for KRAS mutation assessment. **A and B**: Corresponding tissue section with areas marked for macrodissection containing ∼70% tumor cells. Some necrotic areas can not be avoided but in this analogy these do not interfere with DNA extraction. The estimated number of sectioned neoplastic cells in B is ∼4000. For good quality samples, KRAS mutation assessment is reliable with any method, as shown in **C** (sequencing, c. 34 G>A corresponding to the G12S change), in **D** (TMGB-KRAS, G12S mutation positive) and in **E** (DxS-KRAS, G12S mutation positive). DNA control Ct values <29 (arrow) were yielded with both real time PCR methods (red curves in D and E).

**Figure 3:**
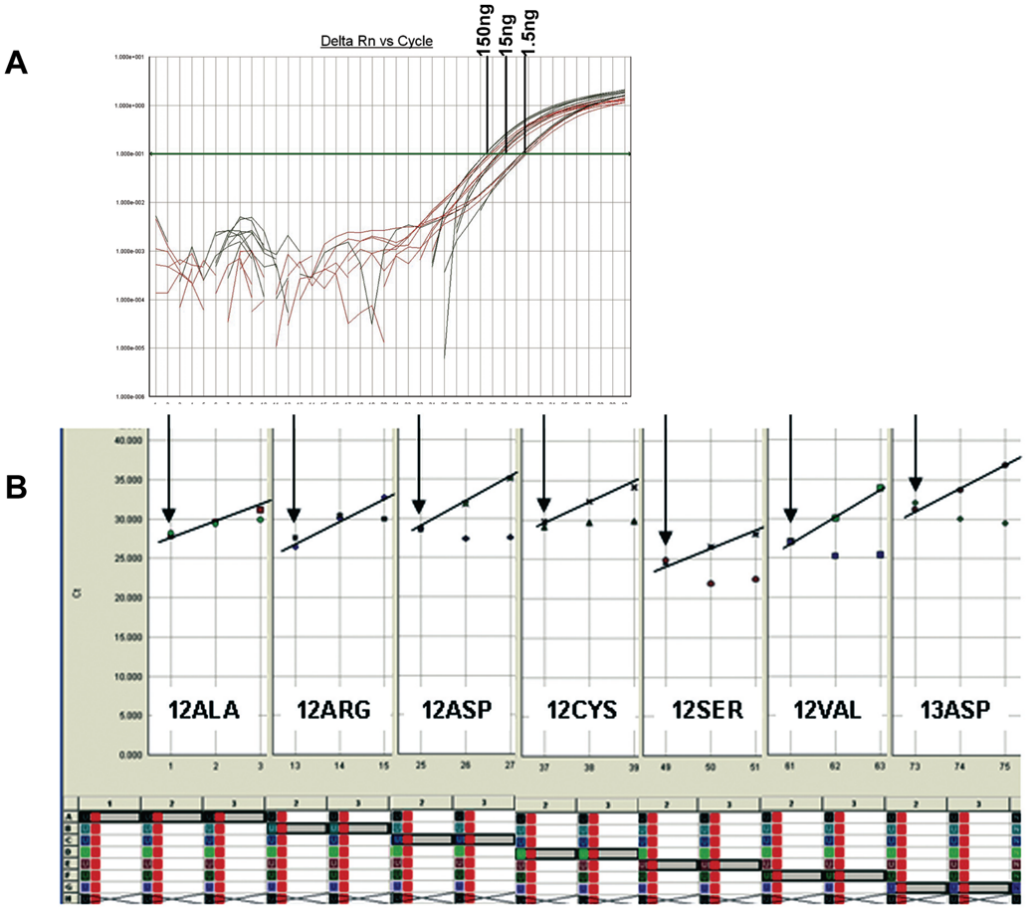
KRAS-TMGB validation on good quality FFPE DNA samples. A. The method can provide reliable results with a broad range of DNA input, as shown here for a G12V mutant sample that was serially diluted up to 1.5 ng/reaction. dCts in all samples are kept close to 0 (safe mutation calling with this method). Red curves, DNA control; Green curves, G12V targeting assay. B. TMGB-KRAS was sensitive to detect <1% tumor cells in good quality FFPE-DNA samples. Seven different mutant FFPE samples containing ∼70% tumor cells (arrows) were diluted at 1∶10 and 1∶100 with different wild-type FFPE-DNA samples. Amplification curve Cts of the diluted mutant DNA showed some degree of linear increase in Ct values for the mutant target, as expected (diagonal lines).

In comparison, samples with less fragmented DNA (favorable DNA quality) performed optimally with all three methods tested in this study (Figure 2). In such samples with tumor cell content >70% (Figure 2B), dCt values with KRAS-TMGB were close to or below 0 (Figure 2D). For such samples, the same dCt values were yielded for a broad range of template input, from 150 ng/reaction down to 1.5 ng/reaction (Figure 3A).

KRAS-TMGB was applied to all samples in groups A and B, yielding informative results in 209/210 cases (99.5%), with only one non-informative sample with very bad DNA characteristics in group B (Table 2). DxS KRAS was mainly employed here to validate the results obtained with KRAS-TMGB in samples where sequencing was non-informative (Tables 1 and 2). DxS-KRAS failed to produce interpretable results in 31.6% of the cases tested, all of them with very fragmented DNA (Table 2). Non-interpretable results were due to either amplification failure of the FAM-DNA control target or Ct values >38. Repeated testing in triplicates for 3 such samples did not improve results.

The above findings show that targeted mutation screening with both real time PCR tests is more efficient than cycle sequencing in identifying mutations in FFPE DNA samples; however, KRAS-TMGB performed significantly better than DxS-KRAS with very fragmented DNA samples (p<0.0001).

### The sensitivity/selectivity of Q-PCR methods may be <1%, but only a minority of diagnostic samples can be analyzed at this level

As described in the Methods section, mutation calling with both DxS-KRAS and KRAS-TMGB is based on the assessment of dCts for the mutant vs control DNA targets. The same parameter is used to assess method selectivity (for example, the requirement for diagnostic methods [21] is sensitivity to detect 1% mutant cells in an environment containing 99 wild-type cells). The lower the percentage of mutant DNA in the sample, the higher are the dCt values obtained and *vice versa*. For example, in order to achieve the desired “1% sensitivity” with DxS, dCt values of ∼9–10 should be assessable. However, to analyze samples at this level of sensitivity, Ct values for the control DNA had to be <29, a condition that was not met for the majority of the samples analyzed.

The results obtained with KRAS-TMGB that was standardized in our lab (Tables 3 and 4) were in the same line. Other than reported previously [42], herein we tested the selectivity of KRAS-TMGB in routine clinical sample conditions, i.e., by serially diluting mutant samples with a high tumor cell content (70%) in KRAS wild-type tumor samples with the same DNA characteristics. For the 1∶10 dilution, a content of <7% tumor cells was anticipated, while for the 1∶100 dilution this content would be <7:1000. The results of this testing are shown in Figure 3B and in Table 4. The finally used cut-off values were assessed based on the dCt values from the 1∶10 dilution assessments (Table 4), that were remarkably close to the ones calculated from all mutant samples (Table 3). According to these cut-off values, 7% mutant tumor cells (or 3.5% mutant alleles) would reliably be identified in samples with wild-type DNA Cts <33, a condition achieved in 95% diagnostic samples and in 1/3 of the very fragmented samples in group B (Table 1). As can also be deduced from Table 4, if working with DNA samples containing >70% tumor cells most of them mutant, it would be safe to analyze samples with wild-type DNA Cts<37. Finally, similar to DxS-KRAS, KRAS-TMGB could detect 0.7% mutant cells with some dCT cut-offs close to 9, as can be deduced from Table 4, again requiring DNA control Cts <29. However, such broad dCts were not observed in any of the 52 samples fulfilling this criterion.

**Table 3:** Assessment of dCt cut-off values for mutation calling with the KRAS-TMGB test in FFPE-DNA samples. Mutant FFPE-DNA samples validated with sequencing.

		dCt mut		
assay	n samples	mean	SD	calculated cut-offˆ
12ALA	5	−0,7132	0,6340	1,1887
12ARG	3	−0,9212	0,4115	0,3132
12ASP	9	0,0646	1,0771	3,2960
12CYS	2	−0,0295	0,9033	2,6804
12SER	3	1,2942	1,2642	5,0868
12VAL	16	0,9986	1,3690	5,1057
13ASP	6	−0,2423	0,8264	2,2368

ˆ  =  mean + 3X SD.

**Table 4:** Detailed assessment of dCt cut-off values for mutation calling with the KRAS-TMGB test. Mutant FFPE-DNA samples* were diluted 1∶10 and 1∶100 in wild-type samples. Correspondingly, final concentrations of 7% and 0.7% tumor cells were anticipated.

	dCt, undiluted		dCt, 1∶10				dCt, 1∶100	
assay	mean	SD	mean	SD	calculated cut-offˆ	cut-off for use	mean	SD
12ALA	−0,0992	0,2284	0,3386	0,2203	0,9997	**<2**	1,2900	0,1531
12ARG	−1,9221	0,0211	−0,1348	0,1863	0,4240	**<2**	1,4814	0,1318
12ASP	−0,3009	0,1802	3,4264	0,1093	3,7543	**4**	7,6930	0,3609
12CYS	0,7338	0,1737	3,5420	0,0616	3,7269	**4**	5,4607	0,1741
12SER	−0,1940	0,1407	4,3250	0,1537	4,7862	**5**	6,3535	0,4833
12VAL	0,4837	0,1256	4,3814	0,1881	4,9458	**5**	6,6008	0,9939
13ASP	−0,6969	0,0817	2,4194	0,1543	2,8823	**3**	4,2344	0,3250

* =  mutant samples, sequencing validated, containing approximately 70% tumor cells.

ˆ  =  mean + 3X SD.

**Figure 4:**
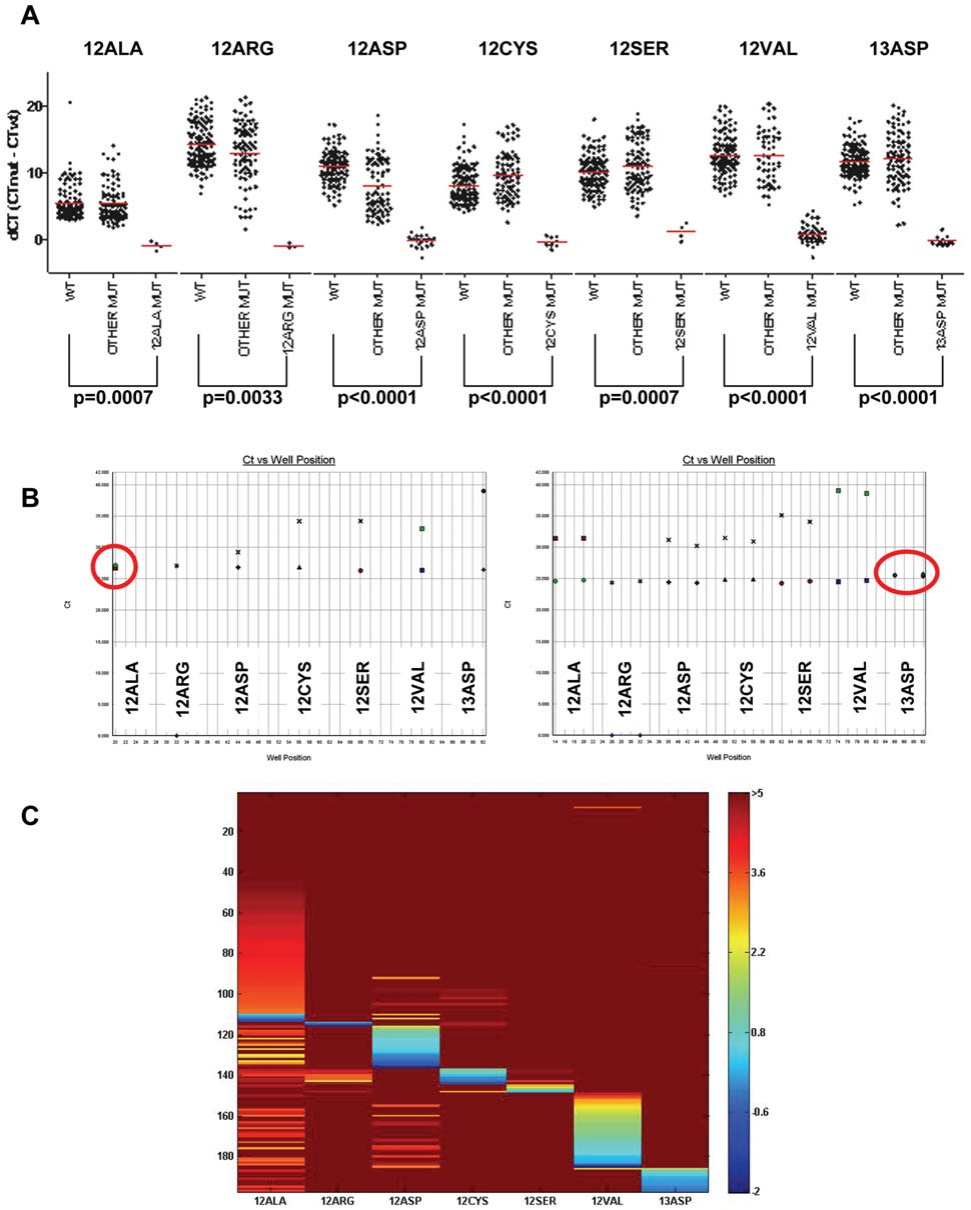
Evaluation of KRAS-TMGB profiles. As shown in **A,** mutant samples have significantly lower dCT values than non-mutant ones. The mutation status of KRAS codons 12 and 13 is evaluated based on the lowest value of the 7-dCt-profile for the corresponding mutant targets, provided that the lowest value falls below the cut-off in each case. In some cases, dCts for the non-mutant alleles can be very low, falling within the range of mutation calling, due to cross-reactivity (labeled as OTHER MUT). Such cross-reactions can be troublesome if evaluating each assay separately. By contrast, if evaluating the profile of dCt values for each sample, as shown in the two examples in **B** with TMGB, the mutant allele can readily be recognized. Here, on the left a sample with G12A, on the right a sample with G13D (duplicates). The dCt profiles of all samples analyzed in this study are shown in the colormap in **C**.

The dCT values for the mutant samples were significantly lower in comparison to the non-mutant samples for each one of the 7 assays of the KRAS-TMGB test (Figure 4A). Evaluating the presence of KRAS mutations with these assays means evaluating the profile of dCts for *all 7 assays simultaneously* for the lowest dCt, which must be lower than the cut-off value for each assay (Figure 4B and C, Table 4). Notably, in some cases dCts close to- or even below the cut-offs were obtained for some of the assays, while the mutant target was clearly distinguishable by a dCt distinctly lower among all 7 values for the same sample. Such cross-reactivity was attributed to increased fluorescence reading from imperfectly matched probes or primers and was observed with DxS-KRAS as well.

### Counting on Q-PCR method sensitivity may yield erroneous results

**Figure 5:**
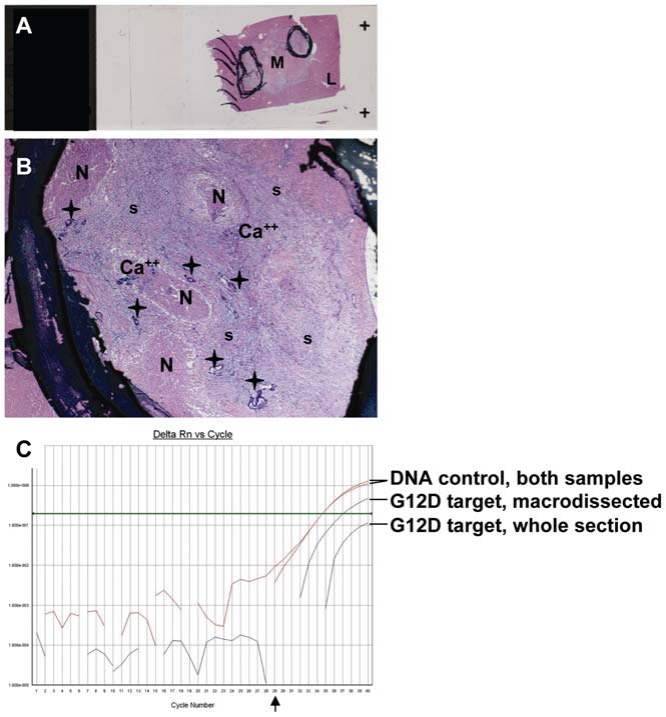
Typical example of a diagnostic case with very low content in neoplastic cells. If macrodissection is avoided in such cases, erroneous results are likely to be obtained. **A**, whole section of the CRC metastatic site (M) surrounded by normal liver (L). Circled areas are marked for macrodissection. In **B**, the metastatic site is largely composed of necrotic (N) and calcified (Ca++) elements within a loose stroma (s), while neoplastic cells (asterisks) correspond to <<1% in the whole section (A) and to ∼10% in the macrodissected areas (B). Two DNA samples were extracted from this specimen, one upon macrodissection and one from the whole section. As shown in **C**, although both DNA samples were of the same unfavorable quality (DNA control Ct∼34.5), it was possible to identify the G12D mutation in the macrodissected sample, while the sample obtained from the whole section appeared as wild type. Arrow in C, Ct = 29.

In 3/135 (2.2%) cases prospectively tested for diagnostic purposes, all from metastatic CRC, we had to perform double extractions from (a) the whole section and (b) upon macrodissection of the neoplastic site, due to reported discrepancies on KRAS mutation status assessed in different labs. Morphologically identifiable tumor cells were <1% in these cases. DNA control Cts varied among 30–36. Whole sections could not be evaluated as positive for KRAS mutations, while samples enriched in tumor cells were mutant (2 Gly12Asp, 1 Gly12Val). One such case is shown in Figure 5.

### Validation of Q-PCR results

Upon external validation rounds for KRAS-TMGB results, matching wild-type, mutation presence and type of mutation were observed in 105/106 cases tested (99,05%). Out of the 66 samples that yielded informative results with the DxS-KRAS-kit, mutations were identified in 29 cases, in full concordance with the corresponding KRAS-TMGB results (100%).

Presence and type of mutations were validated by sequencing in 111 samples. Matching results were initially observed for 109 samples. The two discrepant cases were identified as mutant with the KRAS-TMGB test and initially as wild-type with sequencing. One case was derived from a metastatic CRC site in the lung; upon repeated extraction with further tumor cell enrichment, a heterozygous T substitution could be recognized in the electropherogram. The second case corresponded to a biopsy specimen from the primary site that could not be dissected further and contained <10% tumor cells. This case had a mean DNA control Ct ∼35, yielded a 300bp product with the fragmentation assay, and was evaluated as wild-type with KRAS-TMGB and cycle sequencing in the AUTH lab. However, during external validation, this case was identified as Gly12Asp mutant in the Leuven lab. Upon the 5^th^ attempt, the same DNA sample proved Gly12Asp mutant with cycle sequencing as well. Gly12Asp involves a G:A substitution that can not be regarded as an effect of formalin fixation caused “mutations” as has been reported in such tissues [43]. The difficulty to obtain a proper result with sequencing might be due to the little amount of amplifiable DNA in this case, which should always prompt for careful result evaluation with this method [44].

## Discussion

This study shows that the assessment of DNA fragmentation provides important information on the amplification capacity of the extracted FFPE-DNA and on the reliability of the obtained results, in line with previous reports on methods involving relatively long PCR products [30], [31], [45] but also short ones, as is the case with Q-PCR assays [45]. Based on the degree of DNA fragmentation, FFPE samples can be distinguished into those with relatively well preserved DNA (favorable samples, roughly ¾ of our diagnostic cases) and those with very fragmented DNA (unfavorable samples, ¼ of our diagnostic cases). FFPE-DNA quality depends on numerous, oft imponderable parameters that can not be assessed in the diagnostic setting. For example, tissue block age, a multi-parameter involving at least storage conditions and continuous degradation of nucleic acids after embedding [13], [41], was vaguely related to the degree of DNA fragmentation in our series. If information on storage conditions can not be retrieved, the parameter can not be evaluated. In the same line, type of fixative, time-to-fixation, time-in-fixative, and tissue type are only few of the fixation-related parameters usually addressed for their influence on DNA integrity in FFPE tissues, whereby DNA is better preserved in buffered formalin as compared to simple formalin [16], [43], [46]. Here we can only report on a limited number of biopsy samples (n = 7) that had been fixed overnight in buffered formalin and yielded favorable DNA, as expected. However, since the majority of the favorable DNA samples in this study derived from large colectomy specimens that had been fixed in simple formalin under unknown conditions, fixation in simple formalin can not be blamed as the major determinant of DNA fragmentation.

An intriguing issue concerning FFPE-DNA is to determine the template amount to be added per amplification reaction, which is particularly important for the evaluation of Q-PCR method efficiency. As described, classic UV-measurements (concentration and purity) may be helpful in determining DNA input for PCR in the favorable samples only. In the unfavorable samples, UV-concentration does not correspond to the amount of amplifiable DNA, which is unpredictably low, as indicated by the corresponding high Ct values obtained for control DNA targets (usually >33). This explains why Q-PCR appears dependent on DNA fragmentation, as noticed here in agreement with previous reports [45], [47], [48]. Functional Q-PCR based tests for determining amplifiable DNA quantity are already used in forensic medicine and have been evaluated in FFPE-derived samples as well [47], [48]. It should be noticed though, that DNA degradation may not affect homogeneously all genomic regions [48]. For this reason, and also because commercially available kits for Q-PCR-based DNA quantification still need standardization [49] while increasing cost, it seems important to determine the degree of DNA fragmentation and to identify the unfavorable samples that need special attention in the interpretation of genotyping results by any molecular method.

As depicted in Table 5, all methods evaluated in this study, including classic dideoxy-sequencing, performed optimally with favorable FFPE-DNA; for unfavorable FFPE-DNA however, translating into 1 in 4–5 patients, genotyping results could mostly be obtained by targeted mutation detection with Q-PCR only. This data is fully justified by the small amplicon size used in DxS and TMGB KRAS assays allowing for the amplification of even minimal amounts of preserved amplifiable DNA. Clearly, as per assay design, both DxS-KRAS and KRAS-TMGB can only detect and type 7 mutations in codons 12 and 13, thus probably missing 1% of KRAS mutations in CRC patients. Including assays for KRAS codon 61 substitutions may help to further eliminating this problem.

**Table 5:** Method characteristics of dideoxy-sequencing, DxS-KRAS and KRAS-TMGB in diagnostic KRAS mutation assessment (KRAS codons 12 and 13).

	sequencing	DxS (IVD test)	TMGB
**efficiency**			
relatively well preserved DNA	high	high	high
very fragmented DNA	usually non-informative	high	very high
**accuracy**			
relatively well preserved DNA	golden standard?*	high	high
very fragmented DNA	usually non-informative	high	high
**selectivity**			
relatively well preserved DNA	25–30%	≤1%^&^	<1%
very fragmented DNA	n.a.	usually>10%	usually>10%
**method process**			
method simplicity	complex, multi-step	one-step	one-step
PCR product transfer & handling	required	none	none
time-to-result, post DNA-extraction	days	2 hrs	2 hrs
sample re-processing required	quite oftenˆ	seldom	seldom
**lab experience**			
technical skills required for application	high expertise	basic expertise (PCR)	basic expertise (PCR)
controls	golden standard	provided	sequencing validation required
**cut-offs**	subjective evaluation	provided	assessment in the lab
**cost/DNA sample^#^**	X (sense & antisense)	10X + controls	1,8X + controls

* =  results may not be accurate in cases with tumor cell content below the selectivity of this method, even in samples with preserved DNA

&  =  reported by the manufacturer

ˆ  =  failure to obtain results necessitates troubleshooting at each of the multiple steps of this method; DNA extraction had also to be repeated in some cases

#  =  one successful assessment is meant; cost compared to sequencing with analogies according to reagent prices in Greece

As an IVD-device, DxS-KRAS is a validated and standardized test requiring minimal lab expertise. A concern about such IVD tests is compensating for their high cost, which is partly justified by the stringent standards set for the production of stable reagents. However, the results obtained with this test also depend on template input and setting the reading threshold for obtaining Ct values, two parameters needing further standardization. The reading threshold is of particular importance for assessing Q-PCR sensitivity, as explained below, while it can be set very subjectively when operating with open Q-PCR systems, such as the ABI7500 used here. In comparison, as an in-house developed method, KRAS-TMGB is of low cost but it requires additional lab expertise and efforts for standardization and validation. The specificity and reproducibility of KRAS-TMGB results have been established in this study per sequencing validation, cross-validation with DxS-KRAS and also upon external validation in a different lab. Technically, both Q-PCR methods seem ideal for diagnostic applications (Table 5): minimal hands-on involvement, one-step, one piece of equipment, quick results, easy numeric analysis, easy troubleshooting.

However, even for these highly efficient methods, it is of outmost importance to assess DNA quality (fragmentation and amount of amplifiable DNA) and tumor DNA content for the accurate interpretation of Q-PCR results. DxS-KRAS [http:// www.dxsdiagnostics.com/Content/TheraScreenKRAS.aspx], KRAS-TMGB ([42] and this study), as well as further Q-PCR approaches that have been developed for point mutation assessments [25], [50], may be highly sensitive in detecting rare mutant alleles in a wild type environment. However, this high sensitivity potential is only applicable for samples yielding DNA control Cts below a certain value, usually <29. In our series, most diagnostic FFPE-DNA samples yielded Cts>29 and were, hence, not permissible for analysis at 1% sensitivity. In this regard, it seems necessary to reconsider what 1% sensitivity (or selectivity) stands for when testing FFPE tissues. In samples containing ≥10% tumor cells, detecting 1% mutant cells would correspond to genetic heterogeneity within the same tumor, as previously reported for KRAS in CRCs [28], [29]. Although this may be possible, we did not observe such heterogeneity in any of the 52 cases that could be analyzed at 1% sensitivity in our series. Hence, it seems more pragmatic and helpful to accept cut-offs for lower sensitivity (for example, 10% [Table 5]), so that unfavourable samples can safely be interpreted. Evidently, this condition prompts for tumor cell enrichment and macrodissection. To avoid macrodissection counting on method sensitivity may lead to erroneous results. Clinicians should as well be aware of this shortcoming of the in general supersensitive Q-PCR methods under ideal conditions.

In this study, dideoxy-sequencing was used as a reference for mutation assessment in order to validate the results obtained by Q-PCR assays, especially KRAS-TMGB that had not previously been validated in the diagnostic setting. In this sense, dideoxy-sequencing served its purpose. However, with an efficiency of 82.2%, which may be comparable [24] or higher [23] than reported elsewhere but remains low for a diagnostic test, and with the technical disadvantages described in Table 5, despite the low cost, dideoxy-sequencing can hardly be considered for point mutation detection in the FFPE diagnostic setting. Dideoxy-sequencing still remains the golden standard for DNA analysis, although mutations may be missed in cases with low representation of tumor DNA, as exemplified in two cases in this study. Including as much as possible DNA template for more reliable sequencing results [44] would perhaps help in less fragmented samples, while discrepant results on KRAS mutation status assessed with sequencing and with DxS-KRAS have recently been reported although not defined [51]. Overall, caution is warranted when performing validation trials with dideoxy-sequencing as the golden standard to determine the specificity of methods with higher mutation detection efficiency on FFPE-DNA templates. In comparison to dideoxy-sequencing, pyrosequencing seems to be much more efficient in analyzing 60–80 nt long sequences from FFPE-DNA templates. Pyrosequencing assays for diagnostically relevant KRAS, BRAF and PIK3CA mutations have been developed [52], [53], [54]. Pyrosequencing is simpler, faster and more sensitive than dideoxy-sequencing but, as any sequencing method developed so far, it remains a complex method. This fact along with the required instrumentation and high-lab expertise, may have contributed in the currently limited application of this method in Europe.

Involving both classic histology and molecular testing on FFPE-DNA, the assessment of drug-response-predictive point mutations in solid tumors is a diagnostic practice still in its infancy. If anti-cancer drugs are labelled for specific mutations, robust and reliable diagnostic tests are needed for the identification of the altered genotypes in tumor material. In the case of hot-spot point mutations, as exemplified here with KRAS in CRC, targeted mutation detection with Q-PCR methods appears as the ideal approach. Because these methods are easily applicable (the IVD DxS-KRAS, in particular) and highly efficient, it is expected that their use will spread dramatically, especially if more hot-spot point mutations enter diagnostics [55], [56], [57]. Because, however, FFPE-DNA quality is unpredictable, it is fundamental to consider DNA degradation status and amplification capacity, as well as tumor DNA content in order to interpret Q-PCR results and provide accurate information for clinical use. Failure to embed these parameters in molecular diagnostics may result in erroneous interpretations and ultimately harm clinical oncology practice in the frame of targeted therapy.
